# Reduced Graphene Oxide/Few-Walled Carbon Nanotube Composite Films for Shuttle Suppression in Lithium–Sulfur Batteries: The Role of Surface Functional Groups

**DOI:** 10.1021/acsomega.6c03041

**Published:** 2026-06-08

**Authors:** Danielle D. Justino, Fernanda G. Gandra, Luan T. Cardoso, Rayane C. F. Silva, João Paulo C. Trigueiro, Ana P. C. Teixeira, Marta Sevilla, Rodrigo L. Lavall, Paulo F. R. Ortega

**Affiliations:** † Departamento de Química, 28120 Universidade Federal de Viçosa, Viçosa, Minas Gerais 36570-900, Brazil; ‡ Departamento de Química, 28114 Universidade Federal de Minas Gerais, Belo Horizonte, Minas Gerais 31270-901, Brazil; § Departamento de Química, Centro Federal de Educação Tecnológica de Minas Gerais, Belo Horizonte, Minas Gerais,30421-169, Brazil; ∥ Instituto de Química, 28111 Universidade Federal da Bahia, Salvador, Bahia 40110-909, Brazil; ⊥ Centro de Tecnologia em Nanomateriais e Grafeno da Universidade Federal de Minas Gerais, Universidade Federal de Minas Gerais, Belo Horizonte, Minas Gerais 31310-260, Brazil; # Instituto de Ciencia y Tecnología del Carbono (INCAR), CSIC, Francisco Pintado Fe 26, 33011 Oviedo, Spain

## Abstract

Lithium–sulfur (Li–S) batteries offer high theoretical energy density but are critically limited by the dissolution and migration of soluble polysulfide intermediates, leading to capacity fading and poor cycling stability. In this work, free-standing reduced graphene oxide/few-walled carbon nanotube (rGO@FWCNT) composite membranes are investigated as functional films for Li–S cells, with emphasis on the role of interfacial chemistry. FWCNTs provide mechanical robustness and efficient electronic percolation, while rGO introduces tunable surface functionality and morphology. The composite films exhibit a highly wrinkled, lamellar architecture composed of partially stacked rGO sheets interconnected by a three-dimensional nanotube network, favoring electrolyte infiltration and ionic transport. Despite a decrease in intrinsic rGO conductivity with increasing functionalization, highly functionalized rGO@FWCNT membranes significantly enhance apparent Li^+^ diffusion through surface-assisted diffusion mechanisms mediated by oxygen-containing functional groups. In parallel, these membranes effectively confine soluble polysulfides near the cathode region, suppressing their migration while acting as electrochemically active extensions of the sulfur cathode. As a result, internal resistance, ohmic polarization, and charge-transfer resistance associated with sulfur redox reactions are substantially reduced. Among the investigated systems, the highly functionalized rGO300@FWCNT membrane (24.3 wt % O) delivers the best electrochemical performance, achieving a discharge capacity of 1143 mAh g^–1^ at C/10 along with enhanced rate capability and cycling stability. These results highlight the importance of rational interfacial design in functional membranes for advanced Li–S batteries.

## Graphical abstract

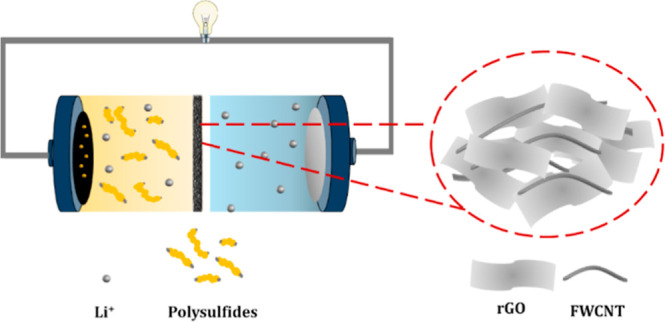

## 1 Introduction

The growing demand for renewable and clean energy sources has intensified efforts toward the development of energy storage devices that are more efficient, sustainable, and economically viable. Currently, lithium-ion batteries (LIBs) dominate the energy storage market; however, their widespread deployment is increasingly challenged by intrinsic limitations, including the approaching limits of achievable gravimetric energy density,^1^ safety concerns associated with flammable organic electrolytes,^2^ and the rising cost and uneven geographical distribution of critical raw materials.^3^ These issues become particularly critical under high-energy and large-scale demand scenarios. In this context, substantial research efforts have been directed toward alternative battery chemistries beyond conventional lithium-ion systems. Among them, lithium–sulfur (Li–S) batteries have emerged as especially attractive candidates due to their exceptionally high theoretical gravimetric energy density of up to 2600 Wh kg^–1^, approximately five times higher than that of commercial LIBs, combined with the low cost, natural abundance, and environmental benignity of sulfur.^4,5^ Owing to these advantages, Li–S batteries are widely regarded as promising energy storage systems for applications in electric vehicles, portable electronics, and large-scale stationary energy storage.^6^

The typical architecture of Li–S batteries consists of a lithium metal anode and a cathode composed of elemental sulfur (S_8_) embedded within carbonaceous matrices,^7^ which are required to provide electronic conductivity due to the intrinsically insulating nature of sulfur. During discharge, metallic lithium is oxidized at the anode, generating Li^+^ ions that migrate through the electrolyte toward the cathode, where sulfur undergoes a sequence of electrochemical reduction reactions, forming, at different stages of discharge, both soluble and insoluble lithium polysulfide species.^8,9^ Despite the high theoretical gravimetric energy density of the Li–S system, the practical energy density achieved at the full-cell level typically corresponds to only 20–35% of this value, mainly due to the additional mass of inactive components such as carbon host materials, current collectors, binders, and the relatively large electrolyte-to-sulfur ratios commonly required for stable operation.^10,11^

Beyond the penalty imposed by inactive components, the most critical obstacle preventing the practical implementation of Li–S batteries arises from the intrinsic reaction mechanism of the system, namely the formation of soluble lithium polysulfides.^12,13^ Once generated at the cathode–electrolyte interface, these species readily dissolve into the electrolyte and migrate through the separator toward the lithium metal anode, where they undergo parasitic reduction reactions.^14,15^ This continuous dissolution–migration–reduction process, commonly referred to as the polysulfide shuttle effect, is widely recognized as the dominant bottleneck limiting the transition of Li–S batteries from laboratory-scale demonstrations to practical devices, as it leads to severe capacity fading, low Coulombic efficiency, and accelerated degradation of the lithium anode.^11,16^ In parallel, the redistribution of sulfur species within the cell can result in the precipitation of insoluble products, which may block the active surface of the cathode or clog the pores of the carbon host, further impairing reaction kinetics.^17^

To mitigate these challenges, several strategies have been extensively explored, including: (i) the rational design of functional cathode materials capable of physically confining or chemically anchoring polysulfide species, thereby suppressing their dissolution;^18^ (ii) electrolyte engineering approaches, such as the use of additives, highly concentrated formulations, or alternative solvent systems that reduce polysulfide solubility and stabilize interfacial reactions;^14,19^ (iii) surface coating and artificial solid–electrolyte interphase (SEI) formation on the lithium metal anode to suppress parasitic reactions and improve interfacial stability;^20^ and (iv) the introduction of functional separators or membranes engineered to restrict polysulfide diffusion toward the anode while maintaining efficient lithium-ion transport.^21,22^ Although each of these approaches has demonstrated partial success, none has been able to fully suppress the shuttle effect without introducing additional complexity, cost, or performance trade-offs at the cell level.

Among the strategies discussed above, the use of functional separators and membranes is particularly attractive from a cell engineering perspective, as it can, in principle, be implemented without major modifications to the fundamental components of the battery and allows straightforward integration into conventional cell configurations. Consequently, the development of functional membranes and separators for Li–S batteries has attracted significant attention in recent years, both in terms of fabrication methodologies and the diversity of materials explored. Commonly employed fabrication approaches include solution-based coating of commercial separators,^23,24^ vacuum-assisted filtration to produce self-supporting membranes,^25^ and layer-by-layer or dip-coating techniques that enable precise control over thickness and surface chemistry.^26^ With respect to material selection, a wide range of functional materials has been investigated, including carbon-based materials such as graphene oxide, reduced graphene oxide, and carbon nanotubes,^27^ polar inorganic compounds (metal oxides, sulfides, and nitrides),^28,29^ polymer-based systems,^30,31^ and porous frameworks such as metal–organic frameworks (MOFs).^32,33^

Among these materials, graphene-based systems and particularly graphene oxide (GO) stand out as some of the most promising candidates for polysulfide-retaining membranes. Current synthesis routes for GO and reduced graphene oxide (rGO) are relatively low-cost and scalable, being largely based on well-established oxidation and acid-assisted exfoliation processes.^29,34^ In addition, these materials exhibit an open lamellar morphology combined with high specific surface area, which is highly favorable for the adsorption of dissolved species.^35,36^ When integrated into or placed in contact with the electrodes, graphene-based materials can also provide sufficient electronic conductivity, which is essential to enable the redox conversion of trapped polysulfides during battery cycling.^37,38^ Despite these advantages and the growing number of studies employing GO-based membranes or separator coatings, several fundamental and sometimes contradictory questions remain unresolved, particularly regarding the role of oxygen-containing functional groups in polysulfide adsorption and overall cell performance.

For instance, Lin et al. reported one of the early and influential studies employing a separator coated with a reduced graphene oxide layer prepared by a doctor-blade process.^39^ Although a clear improvement in electrochemical performance was demonstrated using the functional separator, the authors did not systematically investigate the contribution of surface functional groups, nor did they report their concentration, which is expected to be low due to the high-temperature reduction conditions employed (900 °C under H_2_/Ar atmosphere). In another important reference, Zhu et al. investigated rGO-coated separators with two distinct oxygen contents (15.73 and 37.41 at. %).^40^ In that work, the more reduced rGO exhibited superior electrochemical performance, and the mitigation of the shuttle effect was attributed primarily to polysulfide rejection rather than enhanced chemical adsorption. In contrast, Huang et al. reported improved cell performance using Celgard separators coated with highly functionalized graphene oxide suspensions (oxygen atomic content ≈33%), assigning the positive effect to strong interactions between polar functional groups and polysulfide species.^41^

Graphene and graphene oxide have also been explored in more complex membrane architectures, particularly in the form of composite films.^42^ One representative example was reported by Wang Meng et al., who employed a two-dimensional g-C_3_N_4_/graphene composite as a coating layer on carbon–sulfur electrodes using a slurry-casting approach followed by drying.^43^ In addition to providing electronic conductivity, the authors proposed that both g-C_3_N_4_ and graphene contribute to the chemical binding of long-chain lithium polysulfides. Simpler and highly practical strategies were later introduced by Huang et al.,^41^ and Li et al.,^44^ in which conductive graphene oxide/carbon nanotube (GO/CNT) composite films were fabricated via a facile vacuum-assisted filtration of aqueous dispersions. These free-standing films can be readily detached and directly inserted onto the cathode during coin-cell assembly. In such architectures, CNTs play a dual role by providing continuous electronic pathways while simultaneously forming a porous network that facilitates electrolyte infiltration and lithium-ion transport.

Despite the promising improvements in polysulfide retention and cycling stability reported in these studies, it is increasingly evident that adsorption alone does not govern the overall electrochemical performance of Li–S cells. Instead, multiple physicochemical properties, strongly influenced by the composition and surface chemistry of the graphene oxide employed, act simultaneously. As a result, the lack of systematic and controlled investigations addressing the role of GO functionalization leaves several fundamental questions unresolved, including: (i) how the concentration and nature of oxygen-containing functional groups affect polysulfide adsorption capacity; (ii) how these functional groups influence Li^+^ ion transport across the membrane; and (iii) how changes in electrical conductivity induced by GO functionalization impact contact resistance, charge-transfer processes associated with polysulfide reoxidation, and the overall capacity delivered by the cell.

In this context, the present work aims to advance the state of the art of rGO-based membranes for Li–S batteries by investigating paper-like composite membranes composed of few-walled carbon nanotubes (FWCNTs) and thermally treated graphene oxides with three distinct and well-controlled levels of functionalization (24%, 8%, and 1%). FWCNTs were selected based on prior work from our group, which demonstrated that their high aspect ratio is particularly suitable for the fabrication of buckypapers with excellent electrical conductivity, wettability, and mechanical robustness.^45^ By maintaining a constant conductive framework and systematically varying only the surface chemistry of the rGO component, we evaluate the effect of functionalization on the physicochemical properties of rGO@FWCNT membranes and elucidate how these variations directly translate into key electrochemical parameters of Li–S cells. By clarifying the interplay between surface chemistry and conductive architecture, this study provides rational design guidelines for functional separators with enhanced electrochemical stability, contributing to the development of safer, more efficient, and technologically viable Li–S batteries.

## 2 Experimental Section

### 2.1 Synthesis of the C–S Composite

Polypyrrole was synthesized via oxidative polymerization as described by Sevilla et al.^46^ For this purpose, 7.5 g of pyrrole were added to 500 mL of a 0.5 mol L^–1^ ferric chloride (FeCl_3_) solution. The mixture was kept under constant stirring for 2 h, and the resulting solid (polypyrrole) was separated by filtration, followed by washing with 0.03 mol L^–1^ HCl solution and distilled water until neutral pH was reached. In a subsequent step, 21 g of polypyrrole were physically mixed in a mortar with 2 g of potassium chloride and 5 g of sodium thiosulfate. The mixture was then subjected to thermal treatment under a N_2_ atmosphere, heated to 800 °C at a rate of 10 °C min^–1^, and maintained at this temperature for 1 h. The resulting solid, containing sodium polysulfides (Na_2_S_*x*_), was subsequently dispersed in 500 mL of a 5 mol L^–1^ HCl solution for 8 h under continuous stirring. During this step, inorganic reaction byproducts such as KCl and Na_2_S/Na_2_S_*x*_ were dissolved, while elemental sulfur and H_2_S were formed as a result of the disproportionation of the polysulfides generated during the activation process. The C–S composite was then collected by filtration, washed with distilled water until neutral pH, and dried at 90 °C for 12 h.

### 2.2 Synthesis of Reduced Graphene Oxides

Graphene oxide (GO) was obtained from commercial graphite powder via a modified Hummers oxidation route, as described by Justino et al. ^47^, where 7.5 g of graphite and 7.5 g of NaNO_3_ were mixed with concentrated H_2_SO_4_ under cooling, followed by the gradual addition of 45 g of KMnO_4_. The reaction proceeded at 35 °C for 3 h, after which a 3 wt % H_2_O_2_ solution was added to quench the oxidation. The solid product was washed with deionized water until neutral pH and dried under vacuum at 40 °C. Reduced graphene oxide (rGO) was produced by thermal treatment of GO under nitrogen atmosphere, using a heating rate of 5 °C min^–1^ up to 300, 700, and 1000 °C, with a dwell time of 1 h at each temperature. The resulting samples were denoted rGO300, rGO700, and rGO1000, respectively.

### 2.3 Preparation of the C–S Composite Cathode and rGO@FWCNT Films

The cathode was prepared by dispersing the C–S composite, Super C65 carbon black, and poly­(vinylidene fluoride) (PVDF) in a mass ratio of 80:10:10 using 1-methyl-2-pyrrolidone (NMP) as the solvent. The mixture was stirred at 1000 rpm for 12 h to obtain a homogeneous slurry, which was then cast onto aluminum foil serving as the current collector and dried at 60 °C for 12 h. The coated aluminum foil was cut into circular electrodes with an area of 0.785 cm^2^, resulting in cathodes with an areal mass loading of approximately 1 mg cm^–2^.

For the preparation of the films used for polysulfide retention in the Li–S cells, few-walled carbon nanotubes (FWCNTs) produced at CTNano/UFMG, with diameters ranging from 3 to 8 nm and lengths on the order of 300 μm, were combined with rGOs exhibiting different degrees of functionalization (rGO300, rGO700, and rGO1000). The rGO and FWCNT components were mixed in a 1:1 mass ratio, dispersed in isopropyl alcohol, and sonicated using a probe sonicator for 10 min. The resulting suspensions were vacuum-filtered through a PVDF membrane. After drying for 12 h, the free-standing composite films were carefully peeled off and denoted as rGO300@FWCNT, rGO700@FWCNT, and rGO1000@FWCNT. The resulting free-standing composite films exhibited thicknesses ranging from approximately 0.120 to 0.140 mm.

### 2.4 Polysulfide Retention Experiment Using rGO@FWCNT Films

A lithium polysulfide solution was prepared following the procedure of Rauh et al. by dissolving elemental sulfur (S_8_, 0.6 mol L^–1^) in 60 mL of dimethyl sulfoxide (DMSO) under stirring at 60 °C until a homogeneous solution was obtained.^48^ Lithium was then added in excess to promote sulfur reduction and generate a mixture of lithium polysulfides (Li_2_S_*n*_). Due to the excess lithium, the exact stoichiometry of the polysulfide species could not be determined. Polysulfide formation was qualitatively confirmed by the characteristic color change from blue–green to dark red.

The resulting solution was transferred to an H-type cell, where polysulfide diffusion was evaluated over 36 h using either a conventional separator or rGO@FWCNT-based films. The polysulfide solution was placed in one compartment, allowing diffusion through the separator to assess the polysulfide retention capability of the films under identical conditions.

### 2.5 Physicochemical Characterization of the Materials

Elemental analysis of the reduced graphene oxides (rGOs) was performed using a LECO CHNS-932 microanalyzer, while the total oxygen content was determined using a LECO TF-900 furnace coupled to the same system. X-ray photoelectron spectroscopy (XPS) measurements were carried out on a SPECS spectrometer under a base pressure of 10^–6^ Pa, using a nonmonochromatic Mg Kα X-ray source operated at 12.5 kV and 100 W. The textural properties of rGOs, few-walled carbon nanotubes (FWCNTs), and rGO@FWCNT films were evaluated by N_2_ adsorption–desorption isotherms measured at 77 K using a Micromeritics ASAP 2020 analyzer. The specific surface area was calculated using the Brunauer–Emmett–Teller (BET) method. Prior to analysis, carbonaceous samples were degassed under vacuum at 100 °C for 24 h. Electrical resistance measurements of rGOs and FWCNTs were conducted using the four-point probe method, employing a Keithley 238 high-current source coupled to a four-point probe system. Measurements were performed on pellets prepared with 90 wt % active material (rGOs or FWCNTs) and 10 wt % PTFE binder. The C–S composite cathode was characterized by thermogravimetric analysis (TGA/DTG) under a N_2_ flow of 50 mL min^–1^ at a heating rate of 10 °C min^–1^ using a TGA Q5000 system (TA Instruments). Morphological characterization of the C–S composite and rGO@FWCNT films was performed by scanning electron microscopy (SEM) using FEI Quanta 200 FEG and Thermo Fisher APREO 2 C microscopes. High-resolution images and elemental mapping were obtained by energy-dispersive X-ray spectroscopy (EDS) using an ETD detector operated at 15 kV at the Microscopy Center of UFMG. Fourier-transform infrared (FTIR) spectra were obtained using a Bruker FTIR ALPHA spectrometer equipped with a Platinum ATR module. The measurements were performed in the spectral range from 4000 to 375 cm^–1^, with a resolution of 4 cm^–1^ and 64 accumulated scans for each sample. For the total reflection X-ray fluorescence (TXRF) analysis, a portable S2 PICOFOX spectrometer (Bruker) was used. The equipment is equipped with a molybdenum (Mo) X-ray tube as the excitation source, a multilayer monochromator, and a silicon drift detector (SDD). The membranes were macerated and dispersed prior to analysis. A 1000 mg L^–1^ Sc standard solution was used as the internal standard at a final concentration of 50 mg L^–1^. Additionally, a 4% PVA solution was employed, resulting in a final concentration of 1%, while ultrapure water was used to adjust the final sample volume. Quartz discs with a diameter of 30 mm and thickness of 3.0 ± 0.1 mm were used as sample carriers. To render the quartz surface hydrophobic, 10 μL of a silicone solution in isopropanol (Serva, Germany) was added to the discs prior to sample deposition. Contact angle measurements were performed using an OCA 15EC goniometer from Dataphysics. The membrane samples were directly used as the substrate for the sessile drop measurements. The analyses were carried out using an electrolyte composed of 1 wt % LiNO_3_ in DME/DOL.

### 2.6 Cell Assembly and Electrochemical Measurements

Electrochemical measurements were carried out using CR2032 coin-type cells assembled in an argon-filled glovebox (O_2_ and H_2_O < 0.1 ppm). Two different cell configurations were investigated: reference cells employing a conventional polypropylene separator (Celgard 2500) and modified cells in which rGO@FWCNT films were introduced as functional interlayers.

In all cell configurations, a lithium metal disk (15.6 mm in diameter) was used as the anode, while the sulfur-based composite served as the cathode. Moderate sulfur loading (∼1 mg cm^–2^) and a relatively high electrolyte-to-sulfur ratio (30 μL mg^–1^) were intentionally employed to minimize additional transport limitations and enable a clearer evaluation of the effects associated with membrane surface chemistry and electrochemical behavior.

For the reference cells, a single Celgard 2500 membrane was placed directly between the cathode and the lithium anode. In the modified cells, a free-standing rGO@FWCNT film with a mass loading of approximately 5.11 mg cm^–2^ was inserted between the sulfur cathode and the Celgard 2500 separator, functioning as a cathode-side interlayer.

The electrolyte consisted of a commercial solution of 1 mol L^–1^ lithium bis­(trifluoromethanesulfonyl)­imide (LiTFSI) dissolved in a 1:1 (v/v) mixture of 1,3-dioxolane and 1,2-dimethoxyethane (DOL/DME), containing 1 wt % LiNO_3_ (Shenzhen Capchem Technology). The electrolyte-to-sulfur ratio was fixed at 30 μL mg^–1^ for all cells to allow direct comparison between the different configurations.

Electrochemical measurements were performed using a VMP3 potentiostat/galvanostat (BioLogic). Galvanostatic charge–discharge (GCD) tests were conducted in the voltage range of 1.7–2.7 V (vs Li/Li^+^) at current rates from C/10 to 4 C (C = 1675 mA g^–1^). Cycling stability tests for cells employing rGO@FWCNT functional interlayers were carried out at 0.2 C for up to 500 cycles.

Electrochemical impedance spectroscopy (EIS) measurements were performed at an average potential of 2.21 V versus the Li/Li^+^ reference, using a 5 mV sinusoidal perturbation over a frequency range from 500 kHz to 50 mHz. Cyclic voltammetry (CV) measurements were carried out within the same potential window as the GCD tests at different scan rates.

The lithium-ion diffusion coefficient (D) was estimated from the CV data using the Randles–Sevcík equation^49^

$$I_{p}=2.69\times 10^{5}n^{1.5}AD_{Li^{+}}^{0.5}C_{Li^{+}}v^{0.5}$$

where *I*_p_ (A) is the peak current, *n* is the number of electrons involved in the electrochemical reaction (for Li–S batteries, *n* = 2), *A* (cm^2^) is the effective electrode area (0.785 cm^2^), *C*_Li^+^_ (mol cm^–3^) is the lithium-ion concentration in the electrolyte, and *v* (V s^–1^) is the scan rate. The diffusion coefficient was determined by linearly fitting the peak current (*I*_p_) as a function of the square root of the scan rate (*v* ^0.5^). The diffusion coefficients were calculated using the anodic peak located in the range of 2.4–2.5 V and the two cathodic peaks observed in the ranges of 2.2–2.3 V and 2.0–2.1 V.

## 3 Results and Discussion

### 3.1 Physicochemical Properties of C–S Composite Electrode and GO@FWCNT Films

For the assembly of the Li–S cells, we selected an excellent conductive matrix for sulfur immobilization, as previously reported in the literature. A porous carbon material prepared via the pyrolysis of a mixture containing polypyrrole, KCl, and Na_2_S_2_O_3_ yields sulfur–carbon composites (C–S composites), in which elemental sulfur is generated in situ and incorporated into a N,S-codoped carbon matrix. Under these synthesis conditions, the carbon framework also exhibits a high specific surface area (2467 m^2^ g^–1^) and good electrical conductivity (1.98 S cm^–1^), as previously reported.^46^

To determine the sulfur content resulting from this procedure, thermogravimetric analysis (TGA) was performed under an inert N_2_ atmosphere. Under these conditions, elemental sulfur (S_8_) can be completely removed from the composite without degradation of the carbon matrix. Figure 1A shows the TGA curves of pristine S_8_ and the C–S composite. For pure sulfur, mass loss starts at approximately 160 °C and is completed by 270 °C, corresponding to sulfur sublimation. In contrast, for the composite, these events occur at higher temperatures, with sulfur mass loss starting at around 220 °C and ending at approximately 400 °C. This temperature shift is attributed to stronger van der Waals interactions between S_8_ and the carbon matrix. These effects are more clearly observed in the derivative thermogravimetric (dTGA) curves shown in Figure 1B. The total sulfur content in the composite was determined from the mass loss at 400 °C, corresponding to a sulfur loading of 61 wt %.

**Figure 1:**
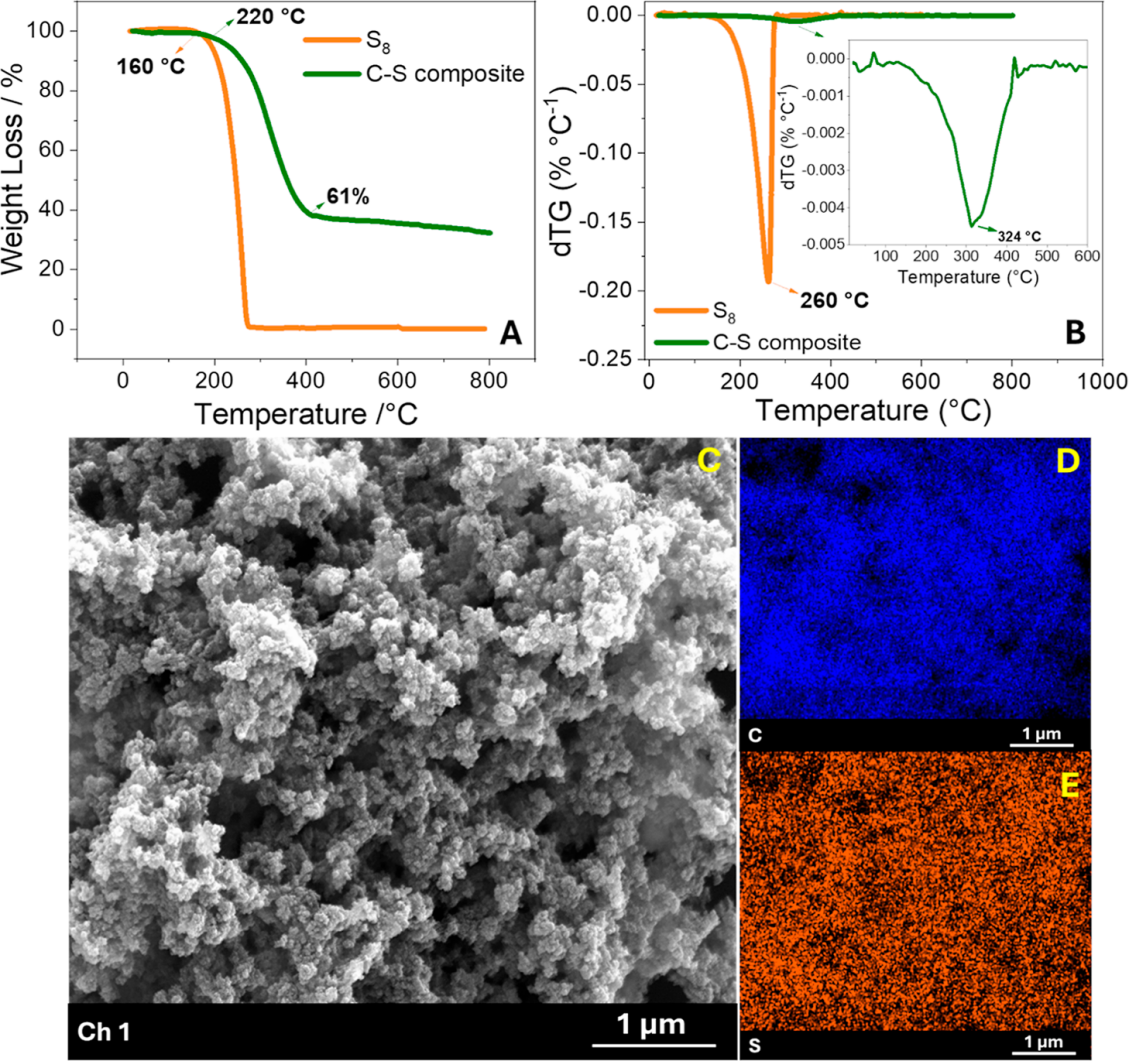
(A) Thermogravimetric (TG) and (B) derivative thermogravimetric (dTG) curves of S_8_ and the C–S composite under a nitrogen atmosphere (flow rate: 50 mL min^–1^). (C) SEM image and corresponding EDS elemental mapping of (D) carbon and (E) sulfur.

The particle morphology and size were further evaluated by SEM, which reveals a sponge-like architecture composed of nanometric aggregates formed by intimately interconnected primary particles, with aggregate diameters ranging from 30 to 80 nm (Figure 1C). The corresponding EDS elemental mapping is also presented, showing a homogeneous spatial distribution of carbon (Figure 1D) and sulfur (Figure 1E) throughout the composite. The uniform overlap between the C and S signals indicates effective sulfur incorporation within the carbon matrix.

The properties and characterization of the rGOs employed in this work for membrane fabrication have been thoroughly discussed in our previous studies.^47,50^ Table 1 summarizes their main features, including elemental composition, the nature and relative abundance of surface functional groups, specific surface area, and electrical conductivity.

**Table 1:** Elemental Composition of the rGO Materials, Relative Contributions of Carbon–Oxygen Bonding Configurations Derived from C 1s XPS Spectra, Specific Surface Area Determined by the BET Method, and Electrical Conductivity Measured Using the Four-Probe Technique^47^

material	specific surface area (m^2^ g^–1^)	C (wt %)	H (wt %)	O (wt %)	–C–O (%) (alcohol)	–C–O (%) (epoxides)	-C=O–(%)	–C(O)OH (%)	electrical resistivity (Ω cm)
rGO300	318	73.4	2.3	24.3	29.0	30.9	28.7	11.4	277.5
rGO700	455	90.1	1.8	8.1	35.8	34.9	23.9	5.3	76.7
rGO1000	435	93.7	1.4	1.3	78.2	0	13.6	8.2	57.0

The rGO materials obtained after acid exfoliation of graphite followed by low-temperature thermal reduction (300 °C) exhibit a high content of oxygen-containing functional groups (24 wt % O), which are relatively uniformly distributed among alcohol, epoxy, and carbonyl functionalities (approximately 30% of each), along with a significant fraction of carboxylic groups (11.4%). Despite the thermal treatment at 300 °C and the nomenclature adopted here for consistency (rGO300), this material should still be regarded as graphene oxide rather than fully reduced graphene oxide. The high density of oxygenated groups is accompanied by a large fraction of sp^3^-hybridized carbon, which significantly disrupts the π-conjugation of the sp^2^ carbon network.^47,51^ As a result, rGO300 exhibits the highest electrical resistivity among the studied samples (277.5 Ω cm).

Upon increasing the reduction temperature to 700 °C, the total amount of oxygen-containing groups decreases by more than half, leading to a substantial reduction in electrical resistivity to 76.7 Ω cm. In the case of rGO1000, the material reaches the highest degree of reduction, with only 1.3 wt % oxygen remaining, predominantly in the form of hydroxyl groups, and exhibits the lowest electrical resistivity (57.0 Ω cm). This highly reduced rGO is therefore the most conductive material and is expected to provide the most efficient electron-transfer pathways to the adsorbed polysulfide species. On the other hand, oxygen-containing functional groups also play a key role in surface wettability, ionic mobility, and the adsorption of polar species, as will be discussed in the following section.

The FWCNT material consists predominantly of double- and triple-walled carbon nanotubes with diameters ranging from 3 to 8 nm and lengths on the order of 300 μm.^45^ This material exhibits a high specific surface area of 402 m^2^ g^–1^ (Figure S1, Supporting Information) and excellent electrical conductivity (1.3 Ω cm), measured in the form of a free-standing buckypaper (Figure S2, Supporting Information). Regarding the rGO300@FWCNT composite films, Figure 2A shows photographic images revealing a paper-like appearance with good mechanical stability. This behavior can be attributed to the interconnected hybrid architecture formed by the rGO sheets and the FWCNT network, in which the nanotubes act as flexible reinforcing elements between adjacent graphene layers, reducing rGO restacking and improving stress distribution throughout the membrane. In addition, residual oxygen-containing functional groups on the rGO sheets, together with van der Waals interactions between rGO and the nanotube surface, contribute to improved interfacial adhesion and structural cohesion. Similar effects have been reported for graphene/CNT hybrid buckypapers and free-standing films.^52–54^ Figure 2B–D present SEM images of the composite films prepared using different rGO materials. No significant morphological differences are observed among them. In all cases, the films exhibit a highly wrinkled and lamellar morphology composed of partially stacked rGO sheets interconnected by a three-dimensional network of carbon nanotubes. The rGO flakes display lateral dimensions in the micrometer range (approximately 3–16 μm), while the FWCNTs act as conductive bridges between adjacent sheets.

**Figure 2:**
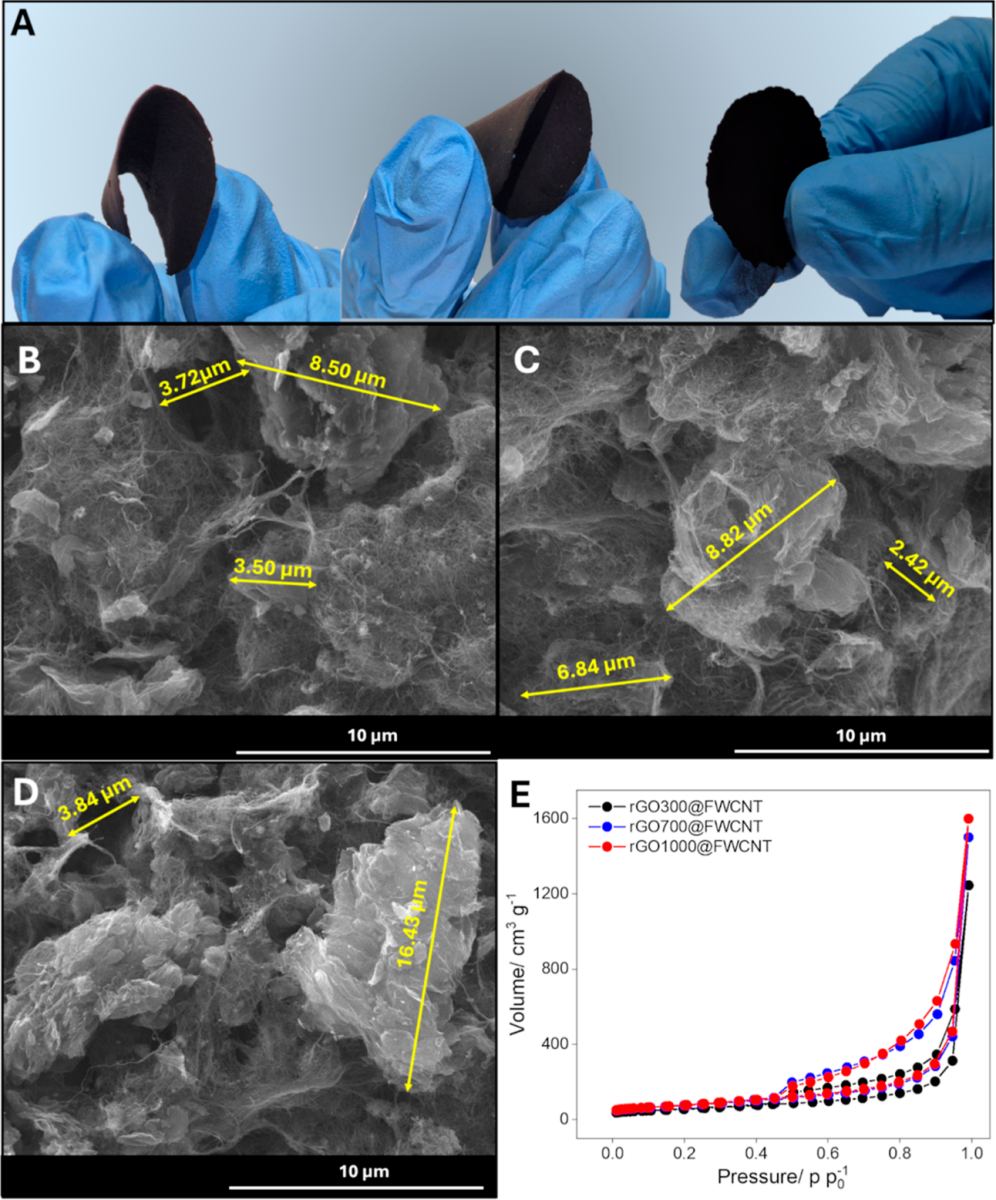
Structural and textural characterization of the rGO@FWCNT films. (A) Photograph of a representative free-standing rGO@FWCNT film. SEM micrographs of (B) rGO300@FWCNT, (C) rGO700@FWCNT, and (D) rGO1000@FWCNT films. (E) N_2_ adsorption–desorption isotherms of the rGO@FWCNT films.

In terms of electrical properties, direct four-point probe measurements of the free-standing rGO@FWCNT membranes revealed a progressive reduction in electronic conductivity with increasing rGO functionalization. The rGO300@FWCNT membrane exhibited the lowest conductivity (2.200 ± 0.313 S·cm^–1^) and highest resistivity (0.461 ± 0.061 Ω·cm), whereas rGO700@FWCNT and rGO1000@FWCNT displayed conductivities of 5.633 ± 0.046 and 10.665 ± 0.468 S·cm^–1^, respectively (Table S1). Despite this decrease in intrinsic electronic conductivity, the highly functionalized rGO300@FWCNT membrane delivered the best electrochemical performance, including lower charge-transfer resistance, higher apparent Li^+^ diffusion coefficients, improved rate capability, and enhanced cycling stability. These results indicate that, within the investigated functionalization range, the beneficial effects associated with oxygen-containing functional groups, such as enhanced polysulfide confinement, improved electrolyte wettability, and facilitated interfacial Li^+^ transport, outweigh the losses in electronic conductivity. The preservation of efficient electronic transport, even in the most functionalized membrane, can be attributed to the interconnected conductive network formed by the FWCNTs, whose high aspect ratio and intrinsic conductivity maintain effective electronic percolation throughout the composite membrane.

The resulting architecture features open interlamellar voids and interconnected channels, which are expected to facilitate electrolyte infiltration and ionic transport across the films. Simultaneously, the continuous FWCNT network enhances electrical percolation throughout the structure, with strong potential to improve overall charge-transport properties. On the other hand, during film formation, the packing and interfacial contact between rGO sheets and FWCNTs lead to a reduction in the specific surface area compared to the individual components. The N_2_ adsorption–desorption isotherms of the rGO@FWCNT membranes are shown in Figure 2E and exhibit a type II profile with H_3_ hysteresis, according to the IUPAC classification.^55^ The specific surface areas, calculated using the BET method, and total pore volumes were 201 m^2^ g^–1^ and 0.485 cm^3^ g^–1^ for rGO300@FWCNT, 272 m^2^ g^–1^ and 0.684 cm^3^ g^–1^ for rGO700@FWCNT, and 283 m^2^ g^–1^ and 0.725 cm^3^ g^–1^ for rGO1000@FWCNT, respectively. The specific surface areas and pore volumes are slightly lower for the films containing more highly functionalized rGOs, which can be attributed to a higher degree of packing at the molecular and nanoscale levels, and partial pore blockage by functional groups. This behavior is likely driven by stronger dipole–dipole and polar interactions arising from the higher density of oxygen-containing functional groups, which promote closer stacking and reduced accessible porosity.

The free-standing rGO@FWCNT composite films exhibited good flexibility and structural integrity during handling and cell assembly. The enhanced mechanical behavior of these membranes can be attributed to the synergistic interaction between reduced graphene oxide (rGO) sheets and functionalized carbon nanotubes (FWCNTs), which promotes the formation of a compact and highly interconnected hybrid network. In this structure, the FWCNTs act as flexible one-dimensional reinforcing elements distributed between the two-dimensional rGO sheets, improving structural cohesion and stress transfer throughout the membrane.

Additionally, the nanotubes prevent excessive restacking of graphene layers while partially filling interlayer voids, resulting in improved structural integrity and resistance to crack propagation. Strong interfacial interactions between oxygen-containing functional groups and the nanotube surface also contribute to improved load-transfer efficiency during mechanical deformation. Similar mechanisms have been widely reported for graphene/CNT hybrid buckypapers and hybrid films, in which the interconnected nanotube–graphene framework enhances tensile strength, flexibility, compactness, and mechanical robustness through efficient stress transfer, suppression of nanotube aggregation, and improved nanotube dispersion.^52,53^

Consistent with these reports, the free-standing rGO@FWCNT membranes developed in this work resisted qualitative bending and torsion tests without visible cracking or fragmentation, demonstrating adequate mechanical robustness for use as flexible cathode-side interlayers in Li–S cells. Although quantitative mechanical measurements were not available in the present work, the qualitative mechanical tests, together with the structural characteristics of the hybrid CNT/rGO architecture and literature reports, strongly support the enhanced mechanical robustness of the free-standing membranes.

Regarding electrolyte wettability of the membranes, contact-angle measurements were attempted using the Li–S electrolyte. However, all membranes showed instantaneous electrolyte absorption which indicates excellent wettability and high electrolyte affinity as showed in the Supporting Information (Video S1).

Before evaluating the effects of incorporating rGO@FWCNT films into Li–S cells, it is essential to assess their ability to adsorb soluble polysulfides. To this end, the membranes were employed as filters in H-type cells, and a concentrated lithium polysulfide (Li_2_S_*n*_) solution prepared in DMSO was placed in one of the reservoirs (right side of the photograph in Figure 3). This configuration ensured a high polysulfide concentration gradient between the two compartments of the cell, as well as an intense coloration to facilitate visual observation. Figure 3 presents photographs recorded over the first 36 h. When a conventional separator is used, polysulfides freely permeate the membrane, and the characteristic dark coloration is clearly observed on the left side of the cell. In contrast, films with a higher density of functional groups, namely rGO300@FWCNT and rGO700@FWCNT, exhibit effective polysulfide retention, with no appreciable change in color. The role of rGO functionalization becomes evident when the rGO1000@FWCNT film is employed. Although more effective than the conventional Celgard 2500 membrane, it does not fully prevent polysulfide transport, which can be detected after 24 h.

**Figure 3:**
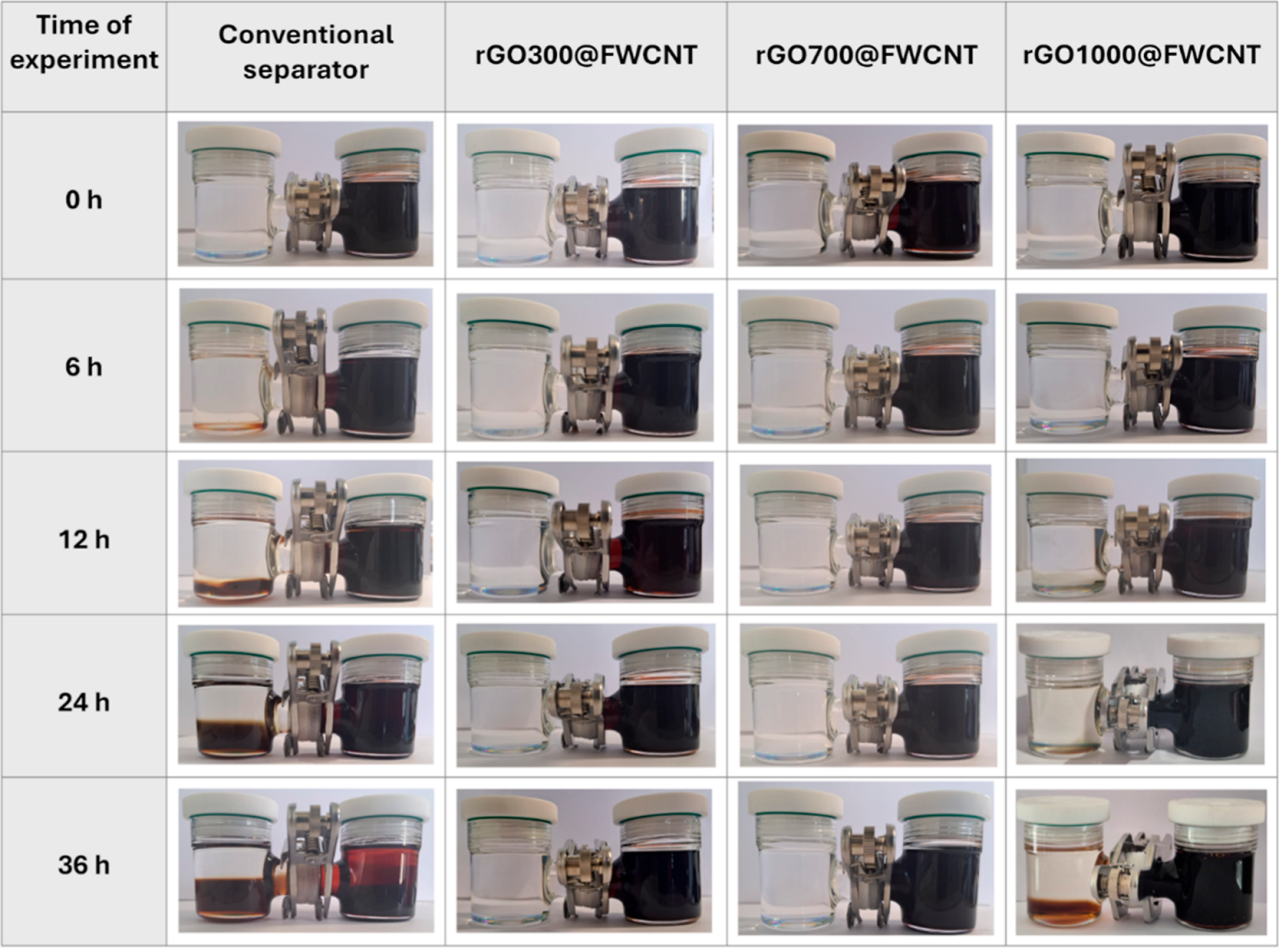
Visual comparison of polysulfide diffusion in H-type cells using a DMSO-based lithium polysulfide solution. From left to right: conventional separator, rGO300@FWCNT, rGO700@FWCNT, and rGO1000@FWCNT films. Photographs taken after the adsorption period illustrate the ability of each membrane to suppress polysulfide migration.

Addionally, studies of the membranes after cycling were carried out by FTIR and TXRF to investigate the role of adsorption of polysulfides. According to FTIR data (Figure S3-Supporting Information), new bands related to sulfur were observed at 863–866 cm^–1^ and 1191–1194 cm^–1^ (S–O); 1047–1055 cm^–1^ and 1132–1136 cm^–1^ (S=O); and 1326–1330 cm^–1^ (SO_2_), indicating the retention of sulfur species by the membranes. The TXRF spectra (Figure S4-Supporting Information) revealed sulfur-related signals associated with retained polysulfide species withing the membranes. The semiquantitative sulfur concentrations (Cs) obtained from TXRF were 123 mg g^–1^ for rGO300@FWCNT, 109 mg g^–1^ for rGO700@FWCNT, and 104 mg g^–1^ for rGO1000@FWCNT. The systematically higher sulfur concentration observed for the more functionalized membranes indicates that oxygen-containing functional groups contribute to retaining sulfur-containing species at the membrane.

### 3.2 Electrochemical Behavior and Performance Evaluation of rGO@FWCNT Films in Li–S Cells

In this section, the electrochemical behavior and performance of Li–S cells are evaluated by comparing systems assembled with a conventional separator and those incorporating rGO@FWCNT films placed on top of the sulfur cathode, as schematically illustrated in Figure 4A.

**Figure 4:**
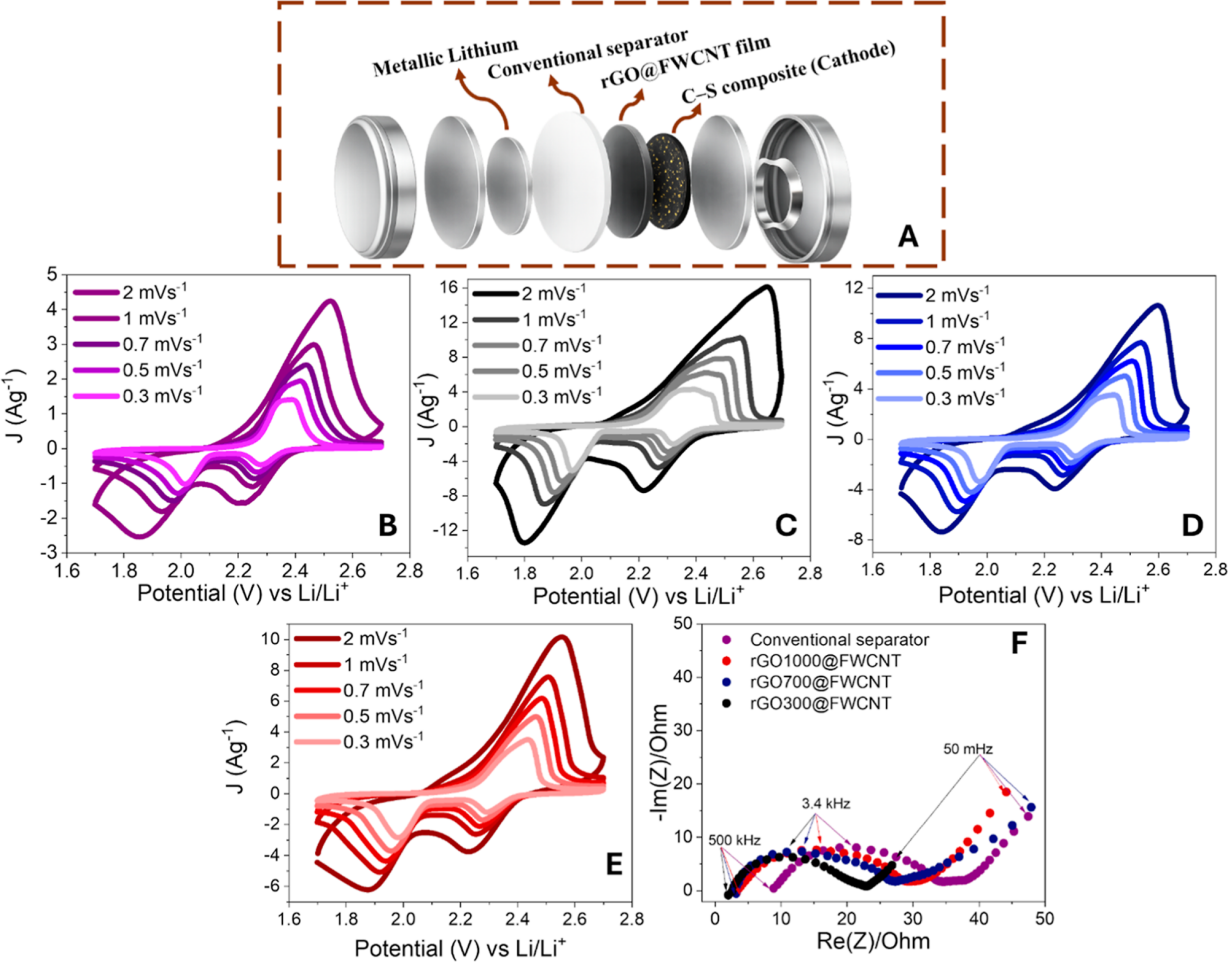
(A) Schematic illustration of the coin cell configuration, with rGO@FWCNT films positioned between the cathode and the conventional Celgard separator. Cyclic voltammetry curves recorded at different scan rates for cells assembled with (B) a conventional separator and with the addition of (C) rGO300@FWCNT, (D) rGO700@FWCNT, and (E) rGO1000@FWCNT films. (F) Nyquist plots obtained from electrochemical impedance spectroscopy (EIS) measurements at an average potential of 2.21 V, over a frequency range from 500 kHz to 50 mHz, using an AC amplitude of 5 mV.

The initial electrochemical investigation was performed using cyclic voltammetry within a potential window of 1.5–2.7 V (vs Li/Li^+^) at scan rates ranging from 0.3 to 2.0 mV s^–1^ (Figure 4B–E). Cyclic voltammetry provides insight into the charge-storage mechanisms of Li–S systems and was also employed here to estimate apparent Li^+^ diffusion coefficients. Overall, the voltammetric profiles remain essentially unchanged upon insertion of the rGO@FWCNT films, indicating that the fundamental sulfur redox pathways are preserved and that the membranes do not alter the intrinsic electrochemical mechanism.

Despite the similarity in peak positions and overall profile shapes, noticeable differences in peak current densities are observed among the cells. Both anodic and cathodic peak currents for the cell equipped with a conventional separator are significantly lower than those observed for cells incorporating the rGO@FWCNT composite films, reflecting variations in the amount of electroactive species participating in the redox processes. This behavior is already due to suppression of polysulfide shuttling and an improvement in reaction kinetics upon insertion of the conductive membranes. Nevertheless, cyclic voltammetry is intrinsically more sensitive to surface-controlled and near-interface processes, whereas quantitative capacity evaluation is more reliably obtained from galvanostatic charge–discharge measurements, discussed later.

In all cases, two characteristic reduction peaks are observed during the cathodic sweep at approximately 2.22 and 1.85 V vs Li/Li^+^. The first peak corresponds to the reduction of elemental sulfur to long-chain lithium polysulfides (Li_2_S_*x*_, 4 ≤ *x* ≤ 8), while the second peak at lower potentials is associated with the further reduction to short-chain lithium polysulfides and insoluble discharge products (Li_2_S_2_ and Li_2_S).^56^ During the anodic scan, two partially overlapping oxidation peaks appear at approximately 2.52 and 2.60 V vs Li/Li^+^, attributed to the oxidation of low-order insoluble polysulfides to higher-order soluble species, followed by the regeneration of elemental sulfur.^57^

Using cyclic voltammograms recorded at different scan rates, apparent Li^+^ diffusion coefficients were estimated from the anodic peak current and the two cathodic peak currents using the Randles–Sevcík equation.^58^ Linearized plots of peak current versus the square root of the scan rate are presented in Figure S5 (Supporting Information), and the calculated diffusion coefficients are summarized in Table 2. It is worth noting that diffusion coefficients derived from Randles–Sevcík analysis in Li–S systems should be regarded as apparent kinetic parameters rather than intrinsic Li^+^ diffusivities in the bulk electrolyte. These values reflect the combined effects of ion transport in confined electrolytes, migration through tortuous porous networks, interactions with dissolved and adsorbed polysulfides, and solid–liquid interfacial charge-transfer kinetics. Importantly, the rGO@FWCNT membrane is not electrochemically inert. Owing to its high electronic conductivity and intimate contact with the sulfur cathode, it acts as an electrochemically active interlayer, effectively extending the reaction zone beyond the conventional cathode–electrolyte interface. Consequently, polysulfide redox reactions mediated by Li^+^ transfer can occur not only at the C–S composite cathode but also at the electrolyte–membrane interface and within the conductive rGO@FWCNT framework.

**Table 2:** Apparent Li^+^ Diffusion Coefficients Determined from CV Analysis for Li–S Cells Using a Conventional Separator and rGO@FWCNT Films

cell configuration	anodic peak (Li_2_S_2_/Li_2_S_(s)_ → Li_2_S_*x*_) D/cm^2^ s^–1^	Cathodic peak 1 (S_8_ → Li_2_S_*x*_) D/cm^2^ s^–1^	Cathodic peak 2 (Li_2_S_*x*_ → Li_2_S_2_/Li_2_S_(s)_) D/cm^2^ s^–1^
Conventional separator	1.06 × 10^–8^	1.58 × 10^–9^	3.00 × 10^–8^
rGO1000@FWCNT	5.68 × 10^–8^	7.89 × 10^–9^	1.34 × 10^–8^
rGO700@FWCNT	1.87 × 10^–7^	2.56 × 10^–8^	5.98 × 10^–8^
rGO300@FWCNT	2.21 × 10^–7^	4.53 × 10^–8^	1.12 × 10^–8^

Within this context, cyclic voltammetry reveals a pronounced enhancement of apparent Li^+^ diffusion upon incorporation of the rGO@FWCNT membranes. For both the anodic process and the first cathodic reduction step, cells containing the conductive membranes exhibit significantly higher diffusion coefficients than the cell employing a conventional separator. Moreover, this enhancement becomes progressively more pronounced with increasing rGO functionalization, following the trend rGO300@FWCNT > rGO700@FWCNT > rGO1000@FWCNT. This behavior is primarily associated with improved electrolyte wettability and ion–surface interactions. At the molecular level, oxygen-containing functional groups can act as temporary coordination sites for Li^+^, enabling a surface-assisted diffusion mechanism.^59,60^ In addition, partial stabilization of intermediate, partially desolvated Li^+^ states by polar functional groups can reduce the effective activation energy for ion transport.^61,62^ Furthermore, suppression of polysulfide lixiviation leads to a higher local concentration of electrochemically active polysulfide species near the cathode region, which also contributes to enhanced apparent Li^+^ diffusion.

In contrast, the second cathodic process associated with the solid-state conversion of soluble polysulfides into Li_2_S_2_/Li_2_S_(s)_ does not follow a monotonic trend. In this case, the rGO700@FWCNT membrane yields the highest apparent diffusion coefficient. A plausible interpretation is that excessive functionalization, as in rGO300@FWCNT, promotes stronger polysulfide interactions and earlier precipitation of solid discharge products, which may partially restrict Li^+^ transport during this solid-state conversion step.

The diffusion coefficients obtained in this work fall within the same order of magnitude as those reported in the literature. For comparison, Venezia et al. reported diffusion coefficients ranging from 2 × 10^–10^ to 2.2 × 10^–11^ cm^2^ s^–1^,^63^ Marangon et al. reported a value of 1.7 × 10^–7^ cm^2^ s^–1^ for the anodic peak,^64^ and Gong et al. reported values between 6.8 × 10^–8^ and 3.32 × 10^–8^ cm^2^ s^–1^,^65^ confirming the consistency of the present analysis.

Further insight into charge-transfer and transport processes was obtained by electrochemical impedance spectroscopy. Figure 4F presents the Nyquist plots for the four investigated cells. All spectra display a depressed semicircle in the high-frequency region followed by an inclined line at low frequencies. The high-frequency intercept on the real axis corresponds to the internal resistance (*R*_s_), which includes contributions from current collectors, electrodes, electrolyte, membranes, and their interfacial contacts. The semicircle in the intermediate-frequency region is associated with the charge-transfer resistance (*R*_ct_) of sulfur redox reactions, while the low-frequency inclined line is related to Li^+^ diffusion in the so-called Warburg region. Notably, insertion of the rGO@FWCNT films leads to a substantial reduction in *R*_s_, from 8.87 Ω for the cell with a conventional separator to 3.52, 3.19, and 1.98 Ω for rGO1000@FWCNT, rGO700@FWCNT, and rGO300@FWCNT, respectively. This behavior has also been reported in previous studies involving rGO-modified separators^66,67^ and can be attributed to the porous architecture and high electronic conductivity of the rGO@FWCNT films, which facilitate electrolyte infiltration and electron transport. The presence of polar oxygen-containing functional groups further enhances electrolyte wettability, explaining the particularly low internal resistance observed for the rGO300@FWCNT membrane. A similar trend is observed for the charge-transfer resistance. *R*_ct_ decreases progressively with increasing rGO functionalization, reaching 20.82 Ω for rGO300@FWCNT, compared to 23.61 Ω, 25.69 Ω, and 26.13 Ω for rGO700@FWCNT, rGO1000@FWCNT, and the conventional separator, respectively. However, this result is not trivial and requires further rationalization. In principle, oxygen-containing functional groups tend to decrease the intrinsic electronic conductivity of rGO and carry negative surface charge, which electrostatically repels anionic polysulfide species. Therefore, such functional groups are not expected to enhance polysulfide adsorption on the membrane. Indeed, Huang et al. reported that electrostatic repulsion was the dominant mechanism responsible for the reduced polysulfide transference across GO membranes.^41^ Nevertheless, the present results clearly show that the incorporation of more highly functionalized rGO leads to a decrease in *R*_ct_, indicating that charge-transfer kinetics are nonetheless enhanced. Similar reductions in *R*_ct_ upon the incorporation of GO-coated separators have also been reported in previous studies.^29,66^ This apparent contradiction can be rationalized by two main factors: (i) suppression of polysulfide lixiviation maintains a higher local concentration of electrochemically active sulfur species near the cathode surface, which favors interfacial reaction kinetics; and (ii) charge transfer can still proceed efficiently at the basal planes of rGO, on the surface of the FWCNTs, and at the C–S composite cathode itself, even in the presence of electrostatic repulsion effects.

The influence of the rGO@FWCNT films on the electrochemical performance of Li–S cells was further evaluated by galvanostatic charge–discharge measurements at current densities ranging from C/10 to 4 C. Representative galvanostatic profiles are shown in Figure 5A–D. In addition to providing gravimetric specific capacities, these curves allow a qualitative assessment of electrode polarization through the voltage gap (Δ*E*) between charge and discharge plateaus. Lower polarization reflects improved electronic conductivity and interfacial compatibility among the C–S composite electrode, the membranes, and the electrolyte.

**Figure 5:**
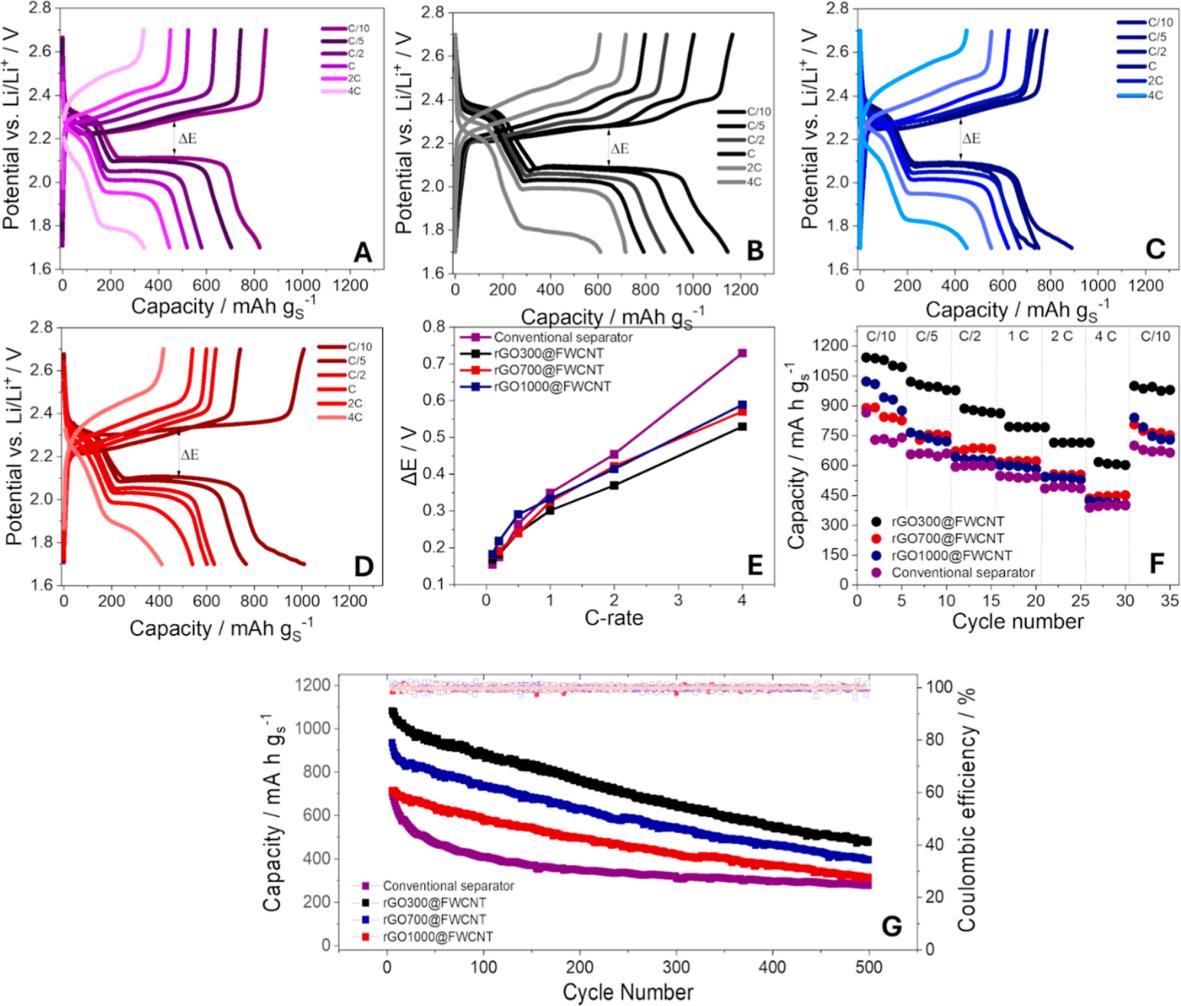
Galvanostatic charge–discharge profiles recorded at different C-rates for cells assembled with (A) a conventional separator and with films composed of (B) rGO300@FWCNT, (C) rGO700@FWCNT, and (D) rGO1000@FWCNT. (E) Voltage polarization (Δ*E*) as a function of the C-rate. (F) Gravimetric specific capacity of the C–S composite at different C-rates for the different cell configurations. (G) Cycling stability at 0.2 C for the different systems.

Since identical C–S composite electrodes were employed in all cells, differences in Δ*E* can be directly attributed to the presence of the rGO@FWCNT films. The polarization values measured at approximately 50% depth of discharge are summarized in Figure 5E. As expected, polarization increases with increasing current density for all cells. However, cells incorporating rGO@FWCNT films exhibit significantly lower Δ*E* values with increasing current density, with the lowest polarization observed for the rGO300@FWCNT membrane. These results are fully consistent with the impedance analysis, which revealed reduced charge-transfer resistance for this system.

The gravimetric specific capacities are also summarized in Figure 5F. The cell with a conventional separator delivers an initial capacity of 866 mAh g^–1^ at C/10, retaining 77% of its capacity after 35 cycles. In contrast, insertion of rGO@FWCNT membranes results in higher capacities at all current densities. The rGO300@FWCNT membrane exhibits the best performance, delivering 1143 mAh g^–1^ at C/10 and 618 mAh g^–1^ at 4 C, with a capacity retention of 86% after 35 cycles at different current densities.

Additionally, the suppression of polysulfide shuttling promoted by the rGO@FWCNT membranes is reflected in the long term cycling stability. Figure 5G shows the cycling performance over 500 cycles at 0.2 C. All cells operate with high Coulombic efficiency, close to 98% throughout cycling. Initial capacities of 1090, 935, 715, and 705 mAh g^–1^ are obtained for cells with rGO300@FWCNT, rGO700@FWCNT, rGO1000@FWCNT, and without film, respectively. After 500 cycles, the remaining capacities are 479, 395, 315, and 278 mAh g^–1^, respectively, confirming the beneficial role of the rGO@FWCNT films, particularly the highly functionalized rGO300@FWCNT, in enhancing the durability and electrochemical performance of Li–S cells. These results are comparable to those reported in the literature for systems employing carbon-based interlayer membranes (Table S2).

Finally, to indirectly evaluate polysulfide shuttle suppression, self-discharge measurements were conducted under open-circuit conditions. The cells were charged to 2.70 V and monitored over time, revealing a clear dependence of voltage decay on membrane composition. After 1 h, the cell with rGO1000@FWCNT exhibited a larger voltage drop (2.42 V), whereas the rGO700@FWCNT and rGO300@FWCNT systems retained higher voltages of 2.55 and 2.57 V, respectively (Figure 6).

**Figure 6:**
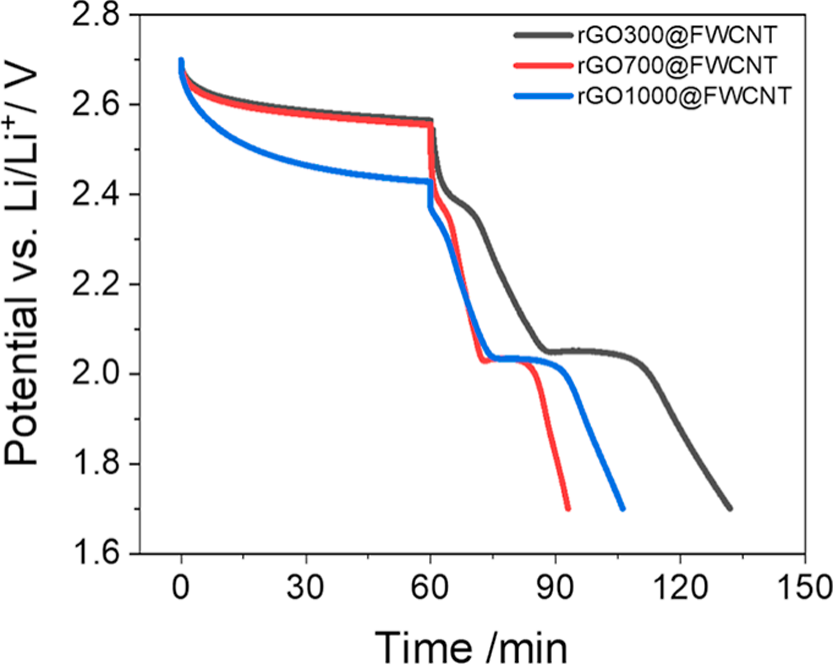
Voltage profiles showing the self-discharge behavior followed by a discharge at 1 C.

The lower voltage decay observed for the more functionalized membranes indicates reduced self-discharge, suggesting more effective polysulfide confinement and smaller capacity loss. In particular, the superior performance of rGO300@FWCNT is attributed to its higher density of oxygen-containing functional groups, which enhances chemical interactions with lithium polysulfides and promotes their retention near the cathode region.

## 4 Conclusion

In this work, we investigated Li–S cells employing free-standing rGO@FWCNT composite films. The incorporation of FWCNTs plays a key role in ensuring the mechanical stability of the films, while also providing high electrical conductivity. The composite films exhibit a highly wrinkled and lamellar morphology, composed of partially stacked rGO sheets interconnected by a three-dimensional carbon nanotube network. This architecture is particularly favorable for electrolyte infiltration and ionic diffusion. In contrast, rGO is the component responsible for tailoring the interfacial chemistry and morphology of the films, thereby modulating the barrier against polysulfide dissolution and migration. In this study, we demonstrate that rGO functional groups not only hinder the transport of soluble polysulfides generated at the cathode, but also perform multiple complementary functions that collectively enhance the electrochemical performance of the Li–S cell.

Although a high degree of functionalization decreases the intrinsic electrical conductivity of the rGO component, we reveal that, within this composite architecture, more highly functionalized rGO@FWCNT films promote enhanced Li^+^ ion diffusion. This behavior arises from oxygen-containing functional groups acting as temporary coordination sites for Li^+^ ions, enabling a surface-assisted diffusion mechanism. Furthermore, despite the electrostatic repulsion between these functional groups and anionic polysulfide species, the membranes are able to confine a higher concentration of polysulfides near the cathode surface. Importantly, the rGO@FWCNT films are not electrochemically inert; instead, they act as an extension of the sulfur cathode, providing additional electrochemically active surface area for sulfur redox reactions. As a consequence, the membranes effectively reduce the internal resistance, ohmic polarization, and charge-transfer resistance associated with sulfur electrochemical processes.

Among the systems investigated, the most highly functionalized membrane, rGO300@FWCNT, composed of rGO containing 24.3 wt % oxygen, exhibited the most pronounced impact on cell performance. Cells employing this membrane displayed lower internal resistance, reduced charge-transfer resistance, the highest discharge capacity (1143 mAh g^–1^ at C/10), and superior cycling stability. Taken together, these findings highlight the critical role of rational interfacial chemistry design in functional membranes and films for Li–S batteries. We anticipate that these results will serve as a guiding framework for the development of advanced membrane-based strategies aimed at suppressing polysulfide shuttling while simultaneously enhancing ion transport and redox kinetics.

## Supplementary Material





## Notes

N_2_ adsorption–desorption isotherms of few-walled carbon nanotubes (Figure S1), DC resistivity measurements of FWCNT buckypapers (Figure S2), FTIR spectra of rGO@FWCNT membranes before and after cycling (Figure S3), TXRF spectra of cycled rGO@FWCNT membranes (Figure S4), Randles–Sevcik plots used for the determination of apparent Li^+^ diffusion coefficients (Figure S5), electrical conductivity and resistivity data of the rGO@FWCNT composite membranes (Table S1), and a comparison of the electrochemical cycling performance of carbon-based interlayer membranes reported in the literature for Li–S batteries (Table S2) (PDF)
A video demonstrating the instantaneous absorption of electrolyte by the membranes (Video S1) (MP4)
