# Supplementary material for: Reduced Graphene Oxide/Few-Walled Carbon Nanotube Composite Films for Shuttle Suppression in Lithium–Sulfur Batteries: The Role of Surface Functional Groups
Source: ACS Omega. 2026 Jun 8;11(24):36008–21. doi: 10.1021/acsomega.6c03041 (PMC13294962; doi:10.1021/acsomega.6c03041)
Supplement: Supplementary file 1 [file ao6c03041_si_001.pdf]

Supporting Information

Reduced Graphene Oxide/Few-Walled Carbon Nanotube Composite Films for Shuttle Suppression in Lithium–Sulfur Batteries: The Role of Surface Functional Groups

Danielle D. Justino[b,c*], Fernanda G. Gandra[b], Luan T. Cardoso[b,e], Rayane C. F. Silva[d], João Paulo C. Trigueiro[c,e], Ana P. C. Teixeira[b,e], Marta Sevilla[f], Rodrigo L. Lavall[b,e], Paulo F. R. Ortega[a*]

[a] Departamento de Química, Universidade Federal de Viçosa, Viçosa, MG 36570-900, Brazil

[b] Departamento de Química, Universidade Federal de Minas Gerais, Belo Horizonte, MG 31270-901, Brazil

[c] Departamento de Química, Centro Federal de Educação Tecnológica de Minas Gerais, Belo Horizonte MG 30421-169, Brazil

[d] Departamento de Química, Universidade Federal da Bahia, Bahia, BA 40110-909, Brazil

[e] Centro de Tecnologia em Nanomateriais e Grafeno da Universidade Federal de Minas Gerais, Universidade Federal de Minas Gerais, Belo Horizonte, MG 31310-260, Brazil

[f] Instituto de Ciencia y Tecnología del Carbono (INCAR), CSIC, Francisco Pintado Fe 26, 33011, Oviedo, Spain

***Corresponding authors**

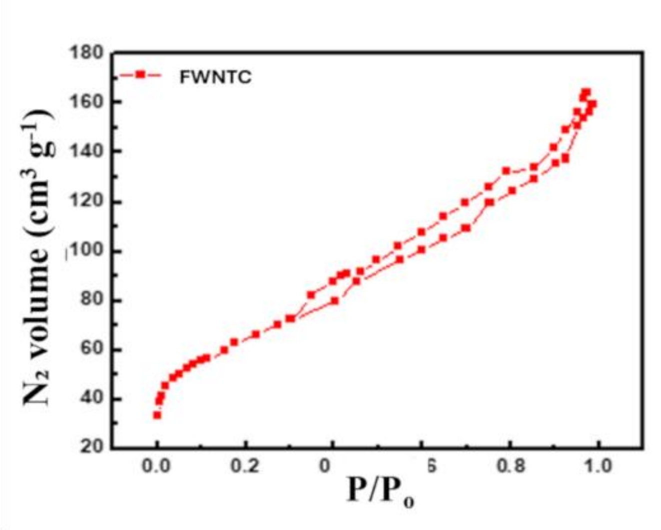

Fig. S1: N_2 adsorption–desorption isotherms of the few-walled carbon nanotubes (FWCNT).

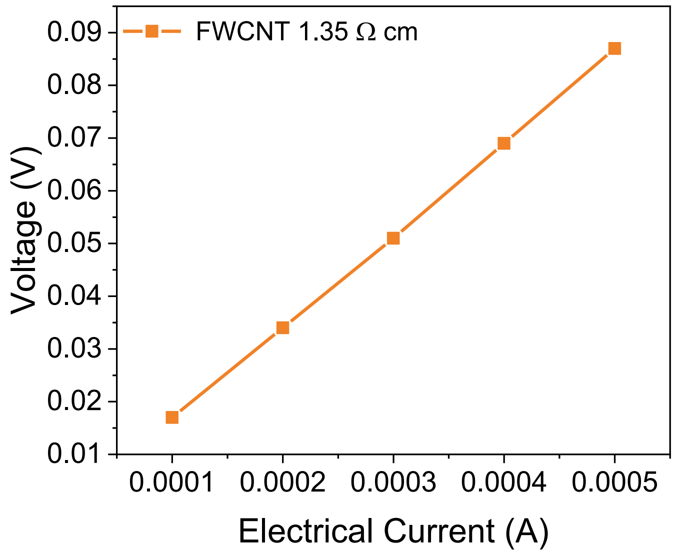

Fig. S2: DC resistivity measurements of few-walled carbon nanotube (FWCNT) buckypapers.

Contact-angle measurements

All membranes showed instantaneous electrolyte absorption which indicates excellent wettability and high electrolyte affinity as showed Video S1.

Video S1: Instantaneous electrolyte absorption by all membranes: https://youtu.be/e-wikoUUKQs?si=6qGBrL72hAgtyYVw

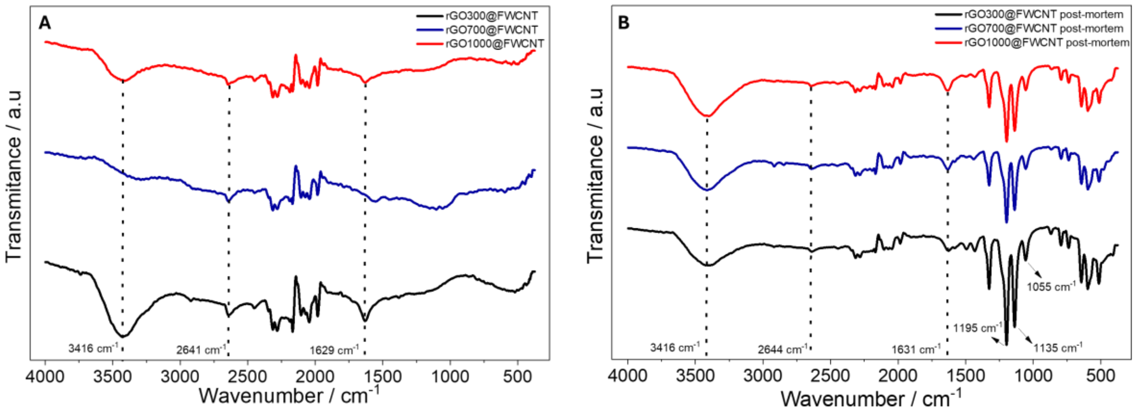

Fig. S3: FTIR spectra of rGO@FWCNT composite membranes with different degrees of rGO functionalization (rGO300@FWCNT, rGO700@FWCNT and rGO1000@FWCNT) before and after cycling.

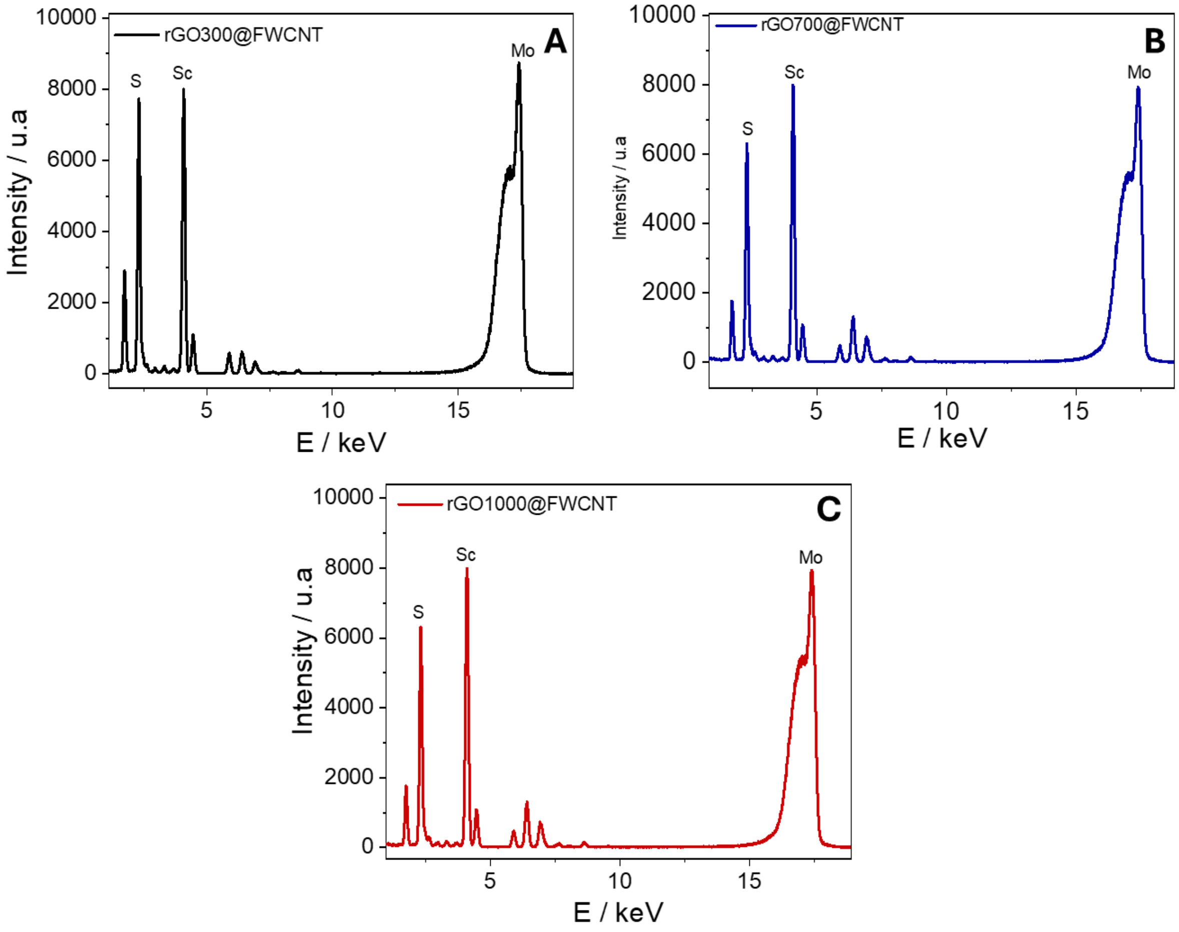

Fig. S4: TXRF spectrum for the rGO@FWCNT membranes after cycling.

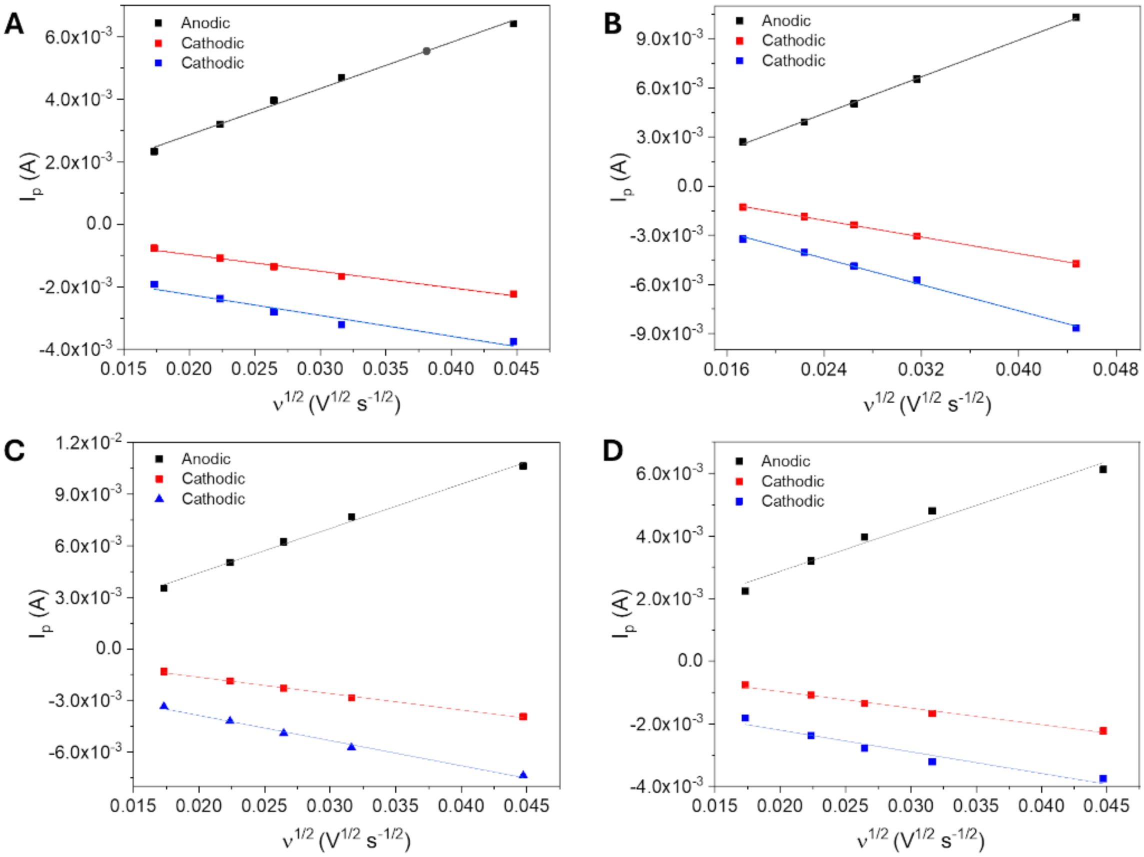

Fig. S5: Plots of peak current versus the square root of the scan rate obtained from cyclic voltammetry for anodic and cathodic reactions in cells assembled with (A) a conventional separator and with rGO@FWCNT films: (B) rGO300@FWCNT, (C) rGO700@FWCNT, and (D) rGO1000@FWCNT.

Table S1: Electrical conductivity, resistivity, and resistance of rGO@FWCNT composite membranes with different rGO functionalization degrees.

Material	Resistivity (Ωcm)	Conductivity (Scm^{-1})
**rGO300@FWCNT**	0.461 ± 0.061	2.200 ± 0.313
**rGO700@FWCNT**	0.178 ± 0.001	5.633 ± 0.046
**rGO1000@FWCNT**	0.094 ± 0.004	10.665 ± 0.468

Table S2: Comparison of electrochemical cycling performance of carbon-based membranes reported for Li–S batteries

Membrane material	Approximate capacity retention	C-rate	Number of cycles	Reference
**rGO300@FWCNT**	63%	0,2C	250	This work
**GO**	65%	0,1C	100	[1]
**MWCNT**	61%	0,2C	150	[2]
**MWCNT**	56%	0,2C	50	[3]
**rGO/Carbon black**	71%	0,1C	100	[4]
**rGO**	72%	2C	250	[5]
**rGO**	70%	1C	400	[6]
**Grafene**	64%	0,2C	100	[7]
**rGO**	60%	0,2C	40	[8]
**rGO**	34%	0,2C	40	[8]

References

(1) Huang, J. Q.; Zhuang, T. Z.; Zhang, Q.; Peng, H. J.; Chen, C. M.; Wei, F. Permselective Graphene Oxide Membrane for Highly Stable and Anti-Self-Discharge Lithium-Sulfur Batteries. *ACS Nano* **2015**, *9* (3).

(2) Chung, S. H.; Manthiram, A. High-Performance Li-S Batteries with an Ultra-Lightweight MWCNT-Coated Separator. *Journal of Physical Chemistry Letters* **2014**, *5* (11).

(3) Su, Y. S.; Manthiram, A. A New Approach to Improve Cycle Performance of Rechargeable Lithium–Sulfur Batteries by Inserting a Free-Standing MWCNT Interlayer. *Chemical Communications* **2012**, *48* (70).

(4) Wang, X.; Wang, Z.; Chen, L. Reduced Graphene Oxide Film as a Shuttle-Inhibiting Interlayer in a Lithium-Sulfur Battery. *J. Power Sources* **2013**, *242*.

(5) Wang, L.; Yang, Z.; Nie, H.; Gu, C.; Hua, W.; Xu, X.; Chen, X.; Chen, Y.; Huang, S. A Lightweight Multifunctional Interlayer of Sulfur-Nitrogen Dual-Doped Graphene for Ultrafast, Long-Life Lithium-Sulfur Batteries. *J. Mater. Chem. A Mater.* **2016**, *4* (40).

(6) Shaibani, M.; Akbari, A.; Sheath, P.; Easton, C. D.; Banerjee, P. C.; Konstas, K.; Fakhfouri, A.; Barghamadi, M.; Musameh, M. M.; Best, A. S.; Rüther, T.; Mahon, P. J.; Hill, M. R.; Hollenkamp, A. F.; Majumder, M. Suppressed Polysulfide Crossover in Li-S Batteries through a High-Flux Graphene Oxide Membrane Supported on a Sulfur Cathode. *ACS Nano* **2016**, *10* (8).

(7) Cengiz, E. C.; Salihoglu, O.; Ozturk, O.; Kocabas, C.; Demir-Cakan, R. Ultra-Lightweight Chemical Vapor Deposition Grown Multilayered Graphene Coatings on Paper Separator as Interlayer in Lithium-Sulfur Batteries. *J. Alloys Compd.* **2019**, *777*.

(8) Zhu, P.; Zang, J.; Zhu, J.; Lu, Y.; Chen, C.; Jiang, M.; Yan, C.; Dirican, M.; Selvan, R. K.; Kim, D.; Zhang, X. Effect of Reduced Graphene Oxide Reduction Degree on the Performance of Polysulfide Rejection in Lithium-Sulfur Batteries. *Carbon N. Y.* **2018**, *126*.
